# Efficient Production of Enterovirus 71 (EV71) Virus-like Particles by Controlling Promoter Strength in Insect Cells

**DOI:** 10.3390/v16060834

**Published:** 2024-05-24

**Authors:** Hyun-Soo Kim, Hyuk-Jin Moon, Jae-Bang Choi, Beom-Ku Han, Soo Dong Woo

**Affiliations:** 1 Department of Agricultural Biology, College of Agriculture, Life & Environment Science, Chungbuk National University, Cheongju 28644, Republic of Korea; 043hsk@gmail.com (H.-S.K.); qlfmzks@chungbuk.ac.kr (H.-J.M.); 2 Optipharm Inc., Osong 28158, Republic of Korea; jbc@optipharm.co.kr (J.-B.C.); bkhan@optipharm.co.kr (B.-K.H.)

**Keywords:** HFMD, enterovirus 71, VLP, baculovirus, burst sequences

## Abstract

This study was conducted to efficiently produce virus-like particles (VLPs) of enterovirus 71 (EV71), a causative virus of hand, foot, and mouth disease (HFMD). The expression level of the P1 precursor, a structural protein of EV71, was modified to increase VLP production, and the optimal expression level and duration of the 3CD protein for P1 cleavage were determined. The expression level and duration of 3CD were controlled by the *p10* promoter, which was weakened by repeated burst sequence (BS) applications, as well as the *OpIE2* promoter, which was weakened by the insertion of random untranslated region sequences of various lengths. The cleavage and production efficiency of the P1 precursor were compared based on the expression time and level of 3CD, revealing that the *p10*-BS5 promoter with four repeated BSs was the most effective. When P1 and 3CD were expressed using the hyperexpression vector and the *p10*-BS5 promoter, high levels of structural protein production and normal HFMD-VLP formation were observed, respectively. This study suggests that the production efficiency of HFMD-VLPs can be significantly enhanced by increasing the expression of the P1 precursor and controlling the amount and duration of 3CD expression.

## 1. Introduction

Enteroviruses are classified into approximately 71 types according to their serotypes. Coxsackievirus A16 is the most common cause of hand, foot, and mouth disease (HFMD), but the most prevalent HFMD virus (HFMDV) in Southeast Asia is enterovirus 71 (EV71) or enterovirus A71 (EV-A71) [1]. In most cases, HFMD is a self-healing disease that presents mild symptoms such as fever and occurs within a week. However, there is a low probability of severe cases in which EV71 invades the central nervous system and causes serious complications, such as meningitis and encephalitis [2]. Complications including hand, foot, and mouth disease are difficult to predict, and no antiviral treatments have been developed. Therefore, no special treatment is available, and most patients improve naturally. However, when symptoms are severe in the acute stage, patients must be hospitalized and endure the symptoms. EV71 infection has a high mortality rate and is primarily responsible for death and morbidity in Southeast Asia [3]. In recent years, EV71 has become the most serious infection affecting children in the Asia–Pacific region. Approximately 80% of severe cases and more than 90% of deaths are caused by EV71. Due to the severity of the virus, an inactivated vaccine was developed by the Institute of Medical Biology of the Chinese Academy of Medical Sciences in 2015 and approved by the Food and Drug Administration (CFDA) of China [4]. The developed vaccine had an efficacy of 97.4%, but it had adverse effects on some patients [4]. Additionally, the inactivated vaccine causes concerning side effects in infants, so a safer and more effective vaccine is needed [5].

Virus-like particle (VLP) vaccines, which aim to minimize the side effects associated with conventional vaccines, are being developed for various viral diseases [6,7]. VLPs closely resemble viruses in appearance but are noninfectious because they do not contain genetic material. VLPs have repetitive, high-density displays of viral surface proteins containing structural viral epitopes that can elicit robust immune responses [8]. Thus, the folding and assembly of recombinant proteins are essential. Compared to common prokaryotic expression systems, eukaryotic expression systems offer more advantages for VLP production [9]. Baculovirus-based insect cell expression systems are commonly used for developing VLP vaccines [10,11,12]. In insect cells, the process of protein production and transformation is often similar to that in humans, and they can produce a variety of VLPs. VLP-type hepatitis B vaccines and HPV vaccines have been commercialized due to their safety and high immunogenicity in humans, and several vaccines have recently been approved [7].

EV71 has nonmembranous particles with a size of 30~32 nm and consists of icosahedral capsids [13]. The viral genome is approximately 7500 nucleotides in length and occurs as +ssRNA with only one open reading frame (ORF). The flanking regions consist of highly structured 5′ UTRs (untranslated regions) and 3′ UTRs with poly(A) tails. The ORF encodes one large polyprotein consisting of 2100 amino acids. The polyprotein is further hydrolyzed to form three precursor proteins: P1, P2, and P3. The P1 precursor protein is cleaved into the following parts: VP1, VP2, VP3, and VP4. Four proteins assemble to form protomers, five protomers form the pentamers, and twelve pentamers form the final virion. VP1, VP2, and VP3 are exposed on the capsid surface, while VP4 is located inside the capsid. P2 and P3 encode seven nonstructural proteins (P2-2A, 2B, 3C, P3-3A, 3B, 3C, and 3D). During the P1 cleavage, the 3CD protease of P3 cleaves between VP0 and VP3, and the 3C protease cleaves between VP3 and VP1 [13]. The mechanism of VP0 cleavage is poorly understood. Therefore, the protein expression of P1 and 3CD must be regulated for HFMD-VLPs, and various expression strategies have been attempted accordingly [12,14,15,16,17,18]. For the first time, the following VLPs generated by co-expressing two types of viruses were reported: P1-expressing viruses generated using the *polyhedrin (polh*) promoter and 3CD-expressing viruses generated using the *p10* promoter [12]. Later, the system was changed to a dual-vector system that simultaneously expresses P1 and 3CD, which can be produced by one virus [12,14,15]. Since then, research has been actively conducted to increase the yield of VLP. In particular, as 3CD has been reported to exhibit cytotoxicity, research is mainly conducted to control the expression level of 3CD [12,16,17,18].

Various promoters are known to express foreign proteins in baculovirus [19]. Among them, the *polh* promoter and the *p10* promoter, which are the most powerful promoters, show excellent efficiency in producing a target protein. To enhance the productivity of the baculovirus expression system, many studies have been conducted to maximize expression efficiency by modifying the promoter [20]. Recently, we reported the construction of a hyperexpression vector that greatly increased protein production efficiency by utilizing hr3, the *p6.9* promoter, and the *polh* promoter and the repeated use of its burst sequences (BSs) [21]. BSs are sequences where a transcription factor, such as Vlf-1 (very late factor-1), is attached and active, and the expression level can be artificially increased by inserting the repeated BSs after the promoter. The *p10* promoter is a very late promoter with the same motif and BS as the *polh* promoter [22]. However, a study showed that the repeated use of BSs proportionally reduced the expression level of the *p10* promoter.

Therefore, to efficiently produce HFMD-VLPs, in this study, we overexpressed the P1 precursor using the hyperexpression vector and determined the expression time and level of the 3CD protein using various promoters, including the *p10* promoter and its BS.

## 2. Materials and Methods

### 2.1. Strategy for HFMD-VLP Production

The overall strategy used to efficiently produce HFMD-VLPs in this study is shown in Figure S1. The parent viral DNA used for recombinant virus generation was MultiBac (Geneva Biotech, Switzerland), which is derived from *Autographa californica* nucleopolyhedrovirus (AcMNPV) DNA lacking the chitinase and cathepsin genes. The hyperexpression vector was used to express the P1 precursor. The very late *p10* promoter and the immediate early *IE2* promoter (*OpIE2*) derived from AcMNPV and *Orgyia pseudotsugata* NPV, respectively, were used to control the expression of 3CD. For the *p10* promoter, the strength was controlled using various repeated BSs and random UTR sequences of various lengths. For the *OpIE2* promoter, the strength was controlled using only UTR sequences of various lengths. The expression efficiency of these promoters was determined using a virus-induced transient expression method [22]. The expression levels of P1 and 3CD by these promoters were determined by comparing the expression levels using previously reported promoters, including *polh*, *p10*, *CMV-IE1*, and baculovirus *gp41* [12,16,18]. The final vector structure for HFMD-VLPs was determined by confirming the level of P1 precursor expression, the level of P1 cleavage by 3CD expression, and VLP production.

### 2.2. Cells and Viruses

The *Spodoptera frugiperda* cell line Sf9 was maintained at 25 °C in an SFM900 II medium (Gibco, Grand Island, NY, USA). The High5 cell line was maintained at 25 °C in Express Five SFM (Gibco, Grand Island, NY, USA) with GlutaMax. rMultiBac generated from MultiBac (Geneva Biotech, Geneva, Switzerland) was used as a control virus. Routine cell culture maintenance and virus production procedures were performed according to published procedures [23].

### 2.3. Virus-Inducible Transient Expression

pHIP vectors containing hr3, the *IE1* promoter, *p10* promoter, and *OpIE2* promoter were used for virus-induced transient expression (Figure S2). To control promoter strength, 1 to 9 repeated BSs of the *p10* promoter or 50 to 400 bp UTR sequences PCR-amplified using λ DNA as a template were placed under the *p10* promoter and the *OpIE2* promoter, respectively. The enhanced green fluorescent protein (EGFP) gene was used as a marker gene to determine promoter strength. Each promoter and marker gene were cloned and used in the experiment. For the intracellular transfer of virus-inducible transient vectors, 0.4 × 10^6^ cultured cells were seeded in a 12-well plate. The transfection of 1.2 µg of DNA into cells was performed using Cellfectin II Reagent™ (Invitrogen, Carlsbad, CA, USA) according to the manufacturer’s instructions. Sixteen hours after DNA transfection, rMultiBac was inoculated at an MOI of 2, and the cells were harvested at 7 days post-infection (p.i.).

### 2.4. Fluorescence Intensity Measurement

To measure the fluorescence intensity of EGFP, virus-infected cells were collected at 1-day intervals and washed with ice-cold PBS. Lysates were prepared via incubation of the cells with 400 µL of lysis buffer (20 mM Tris-HCl, 500 mM NaCl, 1 mM EDTA, 0.1% Tween 20, pH 7.0) and protease inhibitor cocktail (Sigma-Aldrich, Burlington, MA, USA) for 30 min on ice. Fluorescence measurements were performed at room temperature in 96-well plates with a minimum test volume of 100 µL. The fluorescence intensity of the resulting mixture samples was measured using a Synergy HTX Plate Reader (BioTek Inc., Winooski, VT, USA) with an excitation filter of 488 nm and an emission filter of 510 nm. A minimum of three trials were performed, as previously described.

### 2.5. Generation of Recombinant Viruses

Next, the cleavage efficiency of the P1 precursor was evaluated and compared according to the type of promoter used for 3CD expression; His6 tags, which are easy to detect, were attached to both the 5′ and 3′ ends of the P1 precursor (Figure S3A). Recombinant transfer vectors were constructed so that the P1 precursor was expressed by a hyperexpression vector and 3CD was expressed by various promoters. Recombinant viruses were generated using MultiBac, and the recombinant transfer vectors were generated with the Bac-to-Bac system (Invitrogen, Carlsbad, CA, USA). The generated recombinant viruses were named rAcHy-EV-His-P10, rAcHy-EV-His-P10-BS3, rAcHy-EV-His-P10-BS5, rAcHy-EV-OpIE2, rAcHy-EV-His-OpIE2-200, rAcHy-EV-His-OpIE2-400, rAcHy-EV-His-CMV, rAcHy-EV-His-GP41, and rAcHy-EV-His. To determine the final form of the recombinant viruses for HFMD-VLP, His6-tag-removed recombinant viruses were generated using the hyperexpression vector and the standard expression vector using only the *polh* promoter (Figure S3B). The generated recombinant viruses were named rAcPol-EV71-3CD and rAcHy-EV71-3CD.

### 2.6. SDS–PAGE and Western Blot Analysis

Virus-infected cells were harvested by centrifugation at 1000× *g* for 10 min, and the cell pellet was washed with PBS. One hundred microliters of 0.1% PBST (0.1% Triton-X 100 with PBS) was added to the precipitated cells, which were left on ice for 30 min. Next, the lysate was mixed with protein sample buffer and then boiled. The protein samples were subjected to 12% SDS–PAGE. After electrophoresis, the gel was stained with Coomassie brilliant blue solution at room temperature for 1 h and then observed after destaining. For Western blot analysis, SDS–PAGE gels were transferred to nitrocellulose membranes (Pall Corp., Port Washington, NY, USA). The membrane was blocked in 5% skim milk in Tris-buffered saline containing 0.05% Tween 20 and probed with a GFP monoclonal antibody (Abcam, Cambridge, England). The membrane was then incubated with horseradish peroxidase-coupled anti-mouse IgG antibody (Cell Signaling Technology, Danvers, MA, USA), and the bound antibodies were detected using an enhanced chemiluminescence system (Merck Millipore, Burlington, MA, USA) according to the manufacturer’s instructions.

### 2.7. Purification of VLPs

Cells infected with recombinant viruses were harvested at 5 days p.i. and washed with PBS. After 1 mL of PBS (with protease inhibitor) was added to the cell precipitate, cell lysis was performed by the freeze–thaw method, and impurities were removed by filtering with a 0.2 µm syringe filter. The filtrate was carefully dispensed on 10%, 20%, 30%, 40%, and 50% sucrose cushions, and each sucrose fraction was collected by ultracentrifugation at 100,000× *g* and 4 °C for 2 h.

### 2.8. Transmission Electron Microscopy

Negative staining was performed to confirm that virus-like particles formed. After hydrophilicity was induced in the plastic carbon-coated 400 mesh grid using a plasma cleaner, the prepared sample was placed on the grid and left for 5 min, after which the sample droplet was removed using filter paper. To clean the grid, the grid was floated in sterilized water using a 0.2 µm syringe filter and washed three times. Sterile water was removed with filter paper, and uranyless (Electron Microscopy Sciences, Hatfield, PA, USA) was applied onto the grid for negative staining for 1 min. Residual reagents were removed using filter paper. After drying at room temperature for 10 min, the particles were observed using an energy-filtering transmission electron microscope (Carl Zeiss Libera 120) (Zeiss, Oberkochen, Germany).

## 3. Results and Discussion

### 3.1. Determination of 3CD Expression Promoters

**Figure 1:**
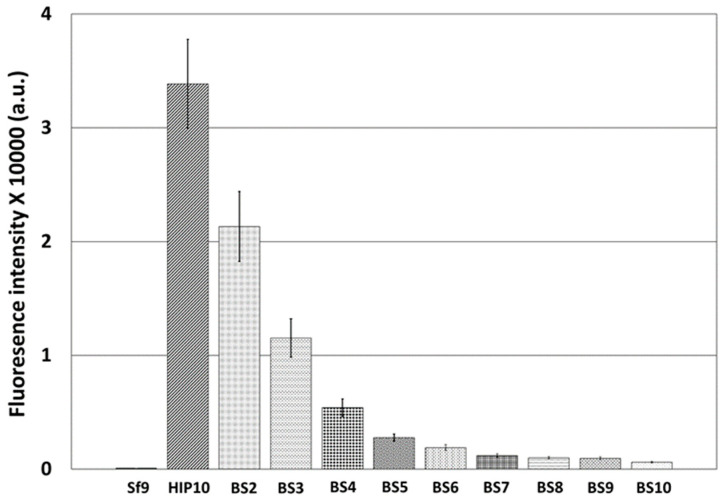
Fluorescence intensities of Sf9 cells by virus-induced transient expression at 7 days p.i. The virus-induced transient expression vectors ranged from 1 (HIP10) to 10 (BS10) BSs of the *p10* promoter downstream of the *p10* promoter. The fluorescence intensity of the cell extracts was measured using a fluorescence spectrometer and is shown in arbitrary units (a.u.). The data are expressed as the mean ± standard error (SE). All experiments were replicated three times.

To determine the optimal expression time and expression level of 3CD for the production of HFMD-VLPs, the strengths of the *p10* promoter and *OpIE2* promoter were compared and evaluated through virus-induced transient expression. To reduce the strength of each promoter, the number of BS repeats or the length of the UTR sequence was varied. For the *p10* promoter of *Bombyx mori* NPV (BmNPV), researchers have reported that promoter strength decreases depending on the repetition of the BS [22]; however, there are no reports on the *p10* promoter of AcMNPV. The effect of repeated BS on the *p10* promoter of AcMNPV was evaluated, and similar to the results in BmNPV, the strength of the *p10* promoter decreased as the number of repeats of *p10*-BS increased (Figure 1). Compared to the expression level observed when the *p10* promoter was used alone, the expression level of BS2 was reduced by approximately 37%, and that of BS3 was reduced by approximately 46%. The reduction rate according to the number of BSs from BS2 to BS10 was approximately 35% on average. BS3 and BS5 reduced the expression levels of these genes by approximately 65% and 90%, respectively.

The burst sequence (BS) is a noncoding sequence of approximately 50–70 bp that occurs within the *polyhedrin* or *p10* promoter of baculovirus and is reportedly necessary for burst expression by these promoters [24]. The p10-BSs of BmNPV and AcMNPV are both 67 bp long and have similar sequences with differences of only five nucleotides. As previously reported, the weakening of the *p10* promoter due to repeated use of the p10-BS of BmNPV results from the uniqueness of the BS sequence [22], and our study also yielded similar results for the AcMNPV *p10* promoter. However, AcMNPV and BmNPV showed some differences in the degree to which the *p10* promoter weakened due to repeated use of p10-BS; however, the overall proportional weakening was identical to that observed with the repeated use of BSs.

**Figure 2:**
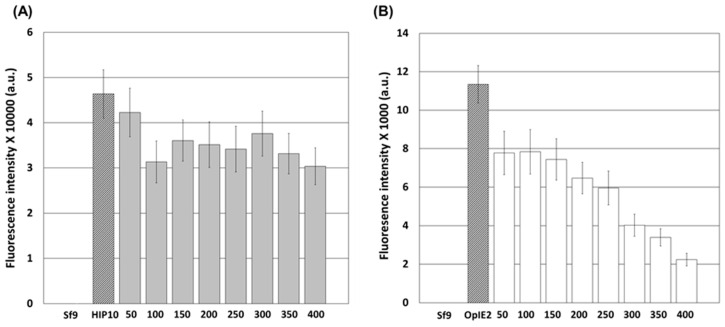
Fluorescence intensities of Sf9 cells after virus-induced transient expression at 7 days p.i. The virus-induced transient expression vectors ranged from 50 bp (50) to 400 bp (400) of the UTR sequences downstream of the *p10* promoter (**A**) and *OpIE2* promoter (**B**). HIP10 and OpIE2 do not have UTR sequences under their respective promoters. The fluorescence intensity of the cell extracts was measured using a fluorescence spectrometer and is shown in arbitrary units (a.u.). The data are expressed as the mean ± standard error (SE). All experiments were replicated three times.

To confirm whether the weakening of the AcMNPV *p10* promoter resulted from BS sequence specificity, as in BmNPV, UTR sequences of various lengths were added behind the *p10* promoter, and the change in strength was evaluated. As a result, when UTR sequences were added behind the AcMNPV *p10* promoter, a decrease in promoter strength was observed, but the correlation between the length of the additional sequences and the promoter strength was not clearly confirmed (Figure 2A). The level of recombinant protein expression by the *p10* promoter was reduced by approximately 34.5% due to the insertion of 400 bp of UTR sequences. Therefore, the decrease in the strength of the *p10* promoter due to the addition of repeated BSs resulted from the specific sequences, not simple sequence addition.

The effect of suppressing downstream sequences is known, in which the strength of the promoter decreases proportionally as the distance between the promoter and the gene increases [25]. However, this effect was not clearly confirmed for the *p10* promoter in this study because the activity of the *p10* promoter was very high, and the effect was not observed when a UTR sequence was added. On the other hand, when a UTR sequence was added behind the *OpIE2* promoter, a decrease in expression intensity was observed depending on the sequence length (Figure 2B). A reduction ranging from approximately 30.9% to 80.2% was observed. The percentages of cells in UTRs-50, 100, and 150 decreased by approximately 31.4%, 30.9%, and 34.4%, respectively, while those in UTRs 200 and 250 decreased by approximately 43.0% and 47.5%, respectively. In addition, 64.6%, 70.1%, and 80.2% of the strains decreased in the UTRs 300, 350, and 400, respectively. Unlike that of the *p10* promoter, the reduction effect due to the addition of the UTR sequence was clearly confirmed for the relatively weak *OpIE2* promoter.

According to the above results, the promoter structures for 3CD expression were BS3, which showed a reduction rate of approximately 46% for the *p10* promoter, and BS5, which showed a reduction rate of approximately 90%. For the *OpIE2* promoter, 3CD expression structures included UTR-200, which showed a reduction rate of approximately 43%, and UTR-400, which showed a reduction rate of approximately 89%.

### 3.2. Expression of HFMDV Structural Proteins

**Figure 3:**
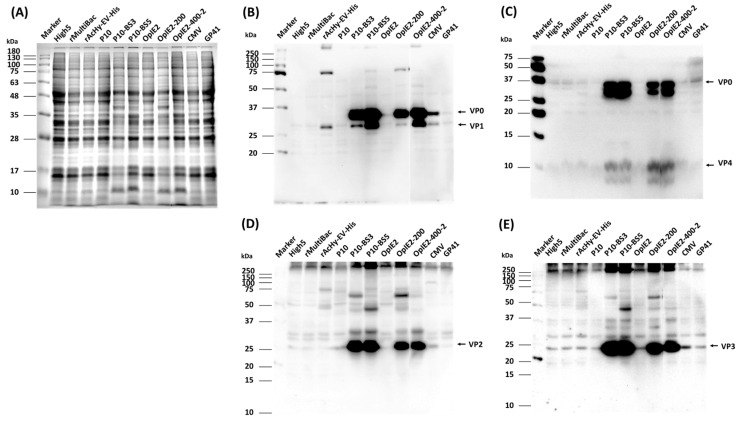
Comparative analysis of cleaved P1 protein production according to the 3CD expression form. Cells were infected with each recombinant virus at a MOI of 5 and harvested 3 days p.i. Protein samples were analyzed by 12% SDS–PAGE (**A**) and Western blot analysis with His6 (**B**), VP4 (**C**), VP2 (**D**), and VP3 (**E**) antibodies.

As a promoter structure for 3CD expression, the previously reported *CMV-IE1* and baculovirus *gp41* promoters were used as controls in addition to the *p10* and *OpIE2* promoters [17,18]. To attain accurate results and easily perform the experiment, the His6-tag was fused to both ends of the P1 precursor and expressed, and the expression level and degree of cleavage of the structural protein were compared and evaluated using VP4 and VP1 as expression indicators. The expression of HFMDV structural proteins by each recombinant virus was confirmed by the presence of an anti-His antibody at approximately 31 kDa for VP1 and approximately 36 kDa for VP0 (Figure 3B). However, the expression of VP4, a cleaved form of the P1, was not detected. The highest levels of expression and cleavage were detected for *p10*-BS3, *p10*-BS5, and *OpIE2*-400. On the other hand, very low levels of expression were observed when the *p10*, *OpIE2*, *CMV-IE1*, and *gp41* promoters were applied. When the *p10* or *OpIE2* promoter was used, its levels were lower than those of the other promoters. These results were reconfirmed using an EV71 antibody. The VP4 antibody produced results similar to those of the anti-His antibody, and the expression of VP4 was confirmed (Figure 3C). Additionally, a specific band presumed to indicate the degradation of VP0 was also observed below VP0. For the VP2 antibody, the expression of a protein of approximately 27 kDa in size, presumed to be VP2, was clearly observed (Figure 3D). The expression level was similar to that shown for anti-His, and the highest expression was observed when the *p10*-BS3 or BS5 promoter was used. The expression of a protein approximately 23 kDa in size, presumed to be VP3, was also detected by the VP3 antibody (Figure 3E). The overall structural protein expression level was similar to that observed for anti-His, and the highest expression was observed when *p10*-BS3 and BS5 were used.

The formation of normal HFMD-VLPs was previously reported when 3CD was expressed by the *p10*, *OpIE2*, *CMV-IE1*, and *gp41* promoters [17,18]. Researchers reported that VLP production was more efficient when the *CMV-IE1* or *gp41* promoter, which is weaker than the *p10* or *OpIE2* promoter, was used as a promoter for 3CD expression. In our study, slightly greater expression was detected when the *CMV-IE1* or *gp41* promoter was used than when the *p10* or *OpIE2* promoter was used. However, compared to all previously reported promoters, the *p10*-BS3 or BS5 promoter led to a much higher expression level, so more efficient HFMD-VLP production was expected. A study using the *gp41* promoter to express 3CD showed that late expression of 3CD is more efficient for VLP production than early expression because 3CD is cytotoxic. In our study, a high expression of structural proteins was detected with the *OpIE2*-200 or *OpIE2*-400 promoter, which had very weak promoter strength; however, the *p10*-BS3 or BS5 promoter appeared to be more efficient.

According to the above results, the strength of the *p10*-BS5 promoter was further weakened; this promoter was identified as the promoter for 3CD expression. Then, a recombinant virus expressing the P1 protein without a His-tag was generated, and VLP analysis was performed.

### 3.3. HFMD-VLP Production

**Figure 4:**
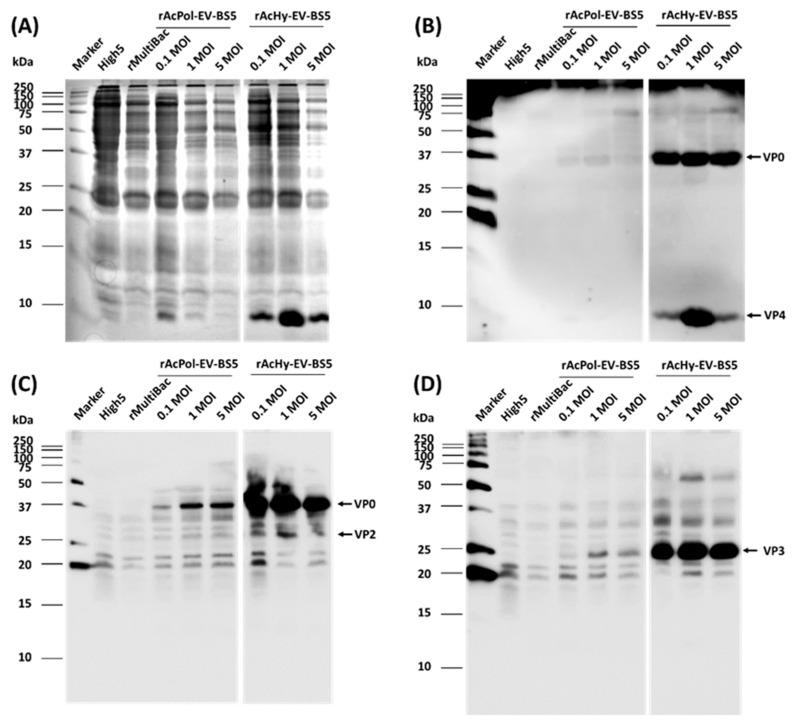
Comparative analysis of cleaved P1 protein production. The P1 precursor was expressed by a hyperexpression vector or standard vector, and 3CD was expressed under the *p10*-BS5 promoter. The cells were infected with each recombinant virus at MOIs of 0.1, 1, and 5 and harvested 3 days p.i. The protein samples were analyzed by 12% SDS–PAGE (**A**) and Western blot analysis with VP4 (**B**), VP2 (**C**), and VP3 (**D**) antibodies, respectively.

**Figure 5:**
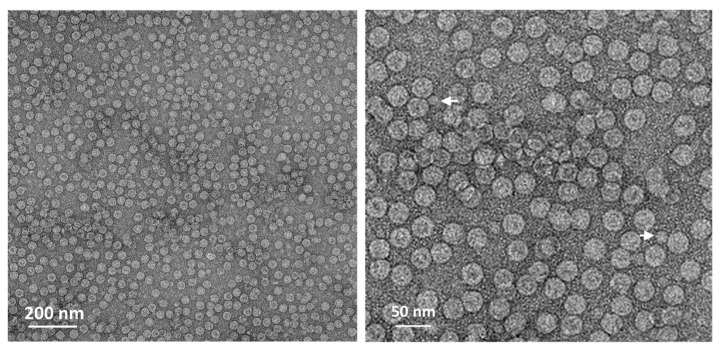
Electron microscopy analysis of HFMD-VLPs. VLP samples were prepared from virus-infected cells via ultracentrifugation sucrose fractionation. Arrows indicate pentamers of HFMDV.

Recombinant viruses expressing 3CD under the *p10*-BS5 promoter and the P1 precursor were generated via the *polh* promoter or hyperexpression vector, respectively. The expression of structural proteins by these recombinant viruses was confirmed, verifying that the expression of structural proteins by the hyperexpression vector was significantly greater (Figure 4). The production of VP4 by VP0 cleavage was clearly observed, but VP0 also remained present (Figure 4B). A virus inoculation dose of 1 MOI was shown to be the most effective. According to these results, the recombinant virus was inoculated into cells, and the formation of HFMD-VLPs was observed at 3 days p.i. Virus-infected cell extracts were observed via transmission electron microscopy after sucrose fractionation, and VLPs with a size of approximately 30–35 nm were successfully formed (Figure 5). In addition, structures presumed to be pentamers other than VLPs were observed in very low amounts, so it was assumed that VLPs were formed in proportion to the expression level of the structural protein.

Compared with vectors generated using only the *polh* promoter, the hyperexpression vectors generated using hr3, the *p6.9* promoter, the *polh* promoter, and repeated BSs have been reported to have approximately 94 times greater expression levels [21]. In this study, the expression level of the HFMDV structural protein in response to the hyperexpression vector was much greater than that in response to the *polh* promoter, so high production of HFMD-VLPs was expected. Additionally, the exact cleavage mechanism of VP0 is not known, and it has been reported that VP4 formation by VP0 cleavage confers stability to the VLP structure [26]. There have also been reports that VLP formation occurs even without VP4. Therefore, although complete cleavage of VP0 was not achieved in our study, the increased production of VP4 or VP0 compared to previous expression forms assessed in our study (Figure 3) indicates the potential for a higher level of VLP production. Further study is needed to clearly confirm this.

To date, various studies have been conducted on HFMD-VLPs [12,14,15,17,18]. In most studies on HFMD-VLP production, the *polh* promoter was used to express the P1 precursor, and the *p10* promoter was used to express 3CD. In addition, it was reported that VLP production can be increased by using a promoter weaker than the *p10* promoter, such as *IE1*, *CMV-IE1*, *gp41*, *lef3*, and the *chitinase* promoter, for 3CD expression [17,18]. To date, research has shown that the production of HFMD-VLPs increases when the *CMV-IE1* or *gp41* promoter is used to express 3CD. Our results showed that the structural protein expression was very high compared to that obtained using the previous *p10*, *CMV-IE1*, and *gp41* promoters. The control of the promoter for 3CD expression may have significantly affected these results; in addition, the use of a hyperexpression vector also greatly increased the expression level of the P1 precursor. However, the *polh* and *p10* promoters used to express the P1 precursor and 3CD, respectively, in our study are known to share many transcription factors. Therefore, these two promoters may mutually influence the strength of these promoters by competitively sharing the transcription factors. In our study, this competition may increase due to the use of repeated BSs. Although polh BS and p10-BS do not share all transcription factors, and transcription factors may be used independently, the *polh* promoter inevitably weakens because the transcription factors are mutually shared. The weakening of the *polh* promoter may cause a decrease in P1 protein production, which may ultimately reduce VLP production. Further study on the mutual influence between these promoters is needed. If the competitive relationship between these two promoters becomes clear through further studies, the production of HFMD-VLPs may be further increased by reselecting the promoter for 3CD expression with low competition to maximize the effect of overexpression of the P1 precursor.

Our study suggests that the efficient production of HFMD-VLPs is possible through quantitative and timely control of 3CD expression along with the overexpression of the HFMDV P1 precursor. We expect that our research results will be useful for producing VLPs from various similar viruses.

## Data Availability

The original contributions presented in the study are included in the article/supplementary material, further inquiries can be directed to the corresponding author.

## Supplementary Materials

The following supporting information can be downloaded at: https://www.mdpi.com/article/10.3390/v16060834/s1, Figure S1: Schematic diagram of the HFMD-VLP production strategy, Figure S2: Schematic representation of the constructed virus-inducible expression vectors, Figure S3: Schematic representation of the recombinant viruses.
