# Supplementary material for: Efficient Production of Enterovirus 71 (EV71) Virus-like Particles by Controlling Promoter Strength in Insect Cells
Source: Viruses. 2024 May 24;16(6):834. doi: 10.3390/v16060834 (PMC11209064; doi:10.3390/v16060834)
Supplement: Supplementary file 1 [file viruses-16-00834-s001.zip › viruses-2999328-supplementary.pdf]

Supplementary Data

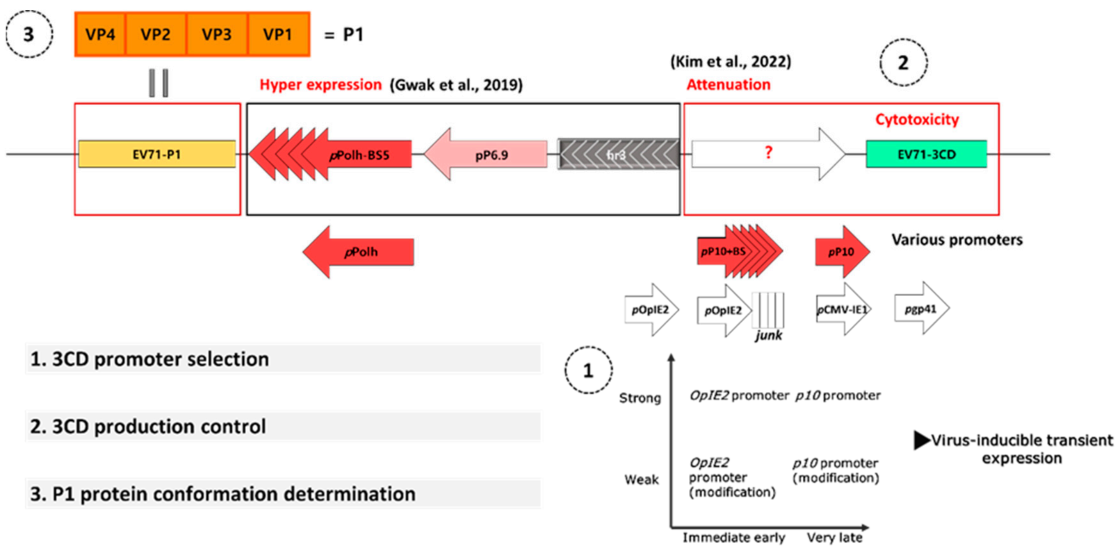

Figure S1. Schematic diagram of the HFMD-VLP production strategy.

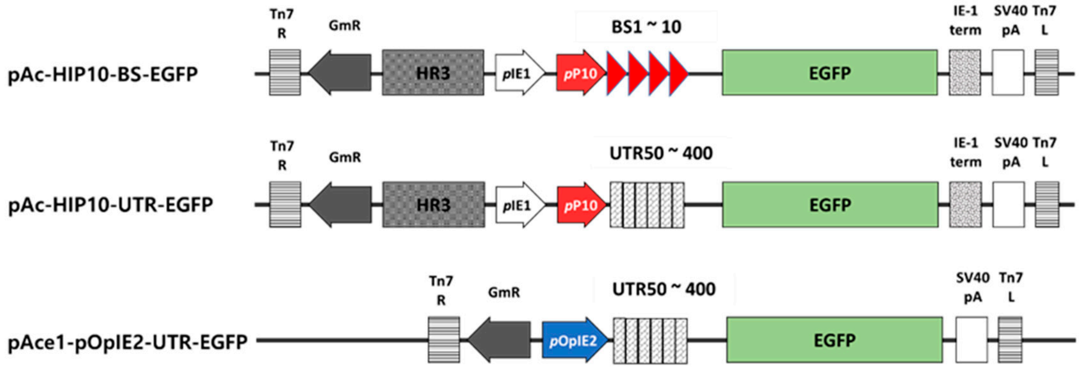

Figure S2. Schematic representation of the constructed virus-inducible expression vectors.

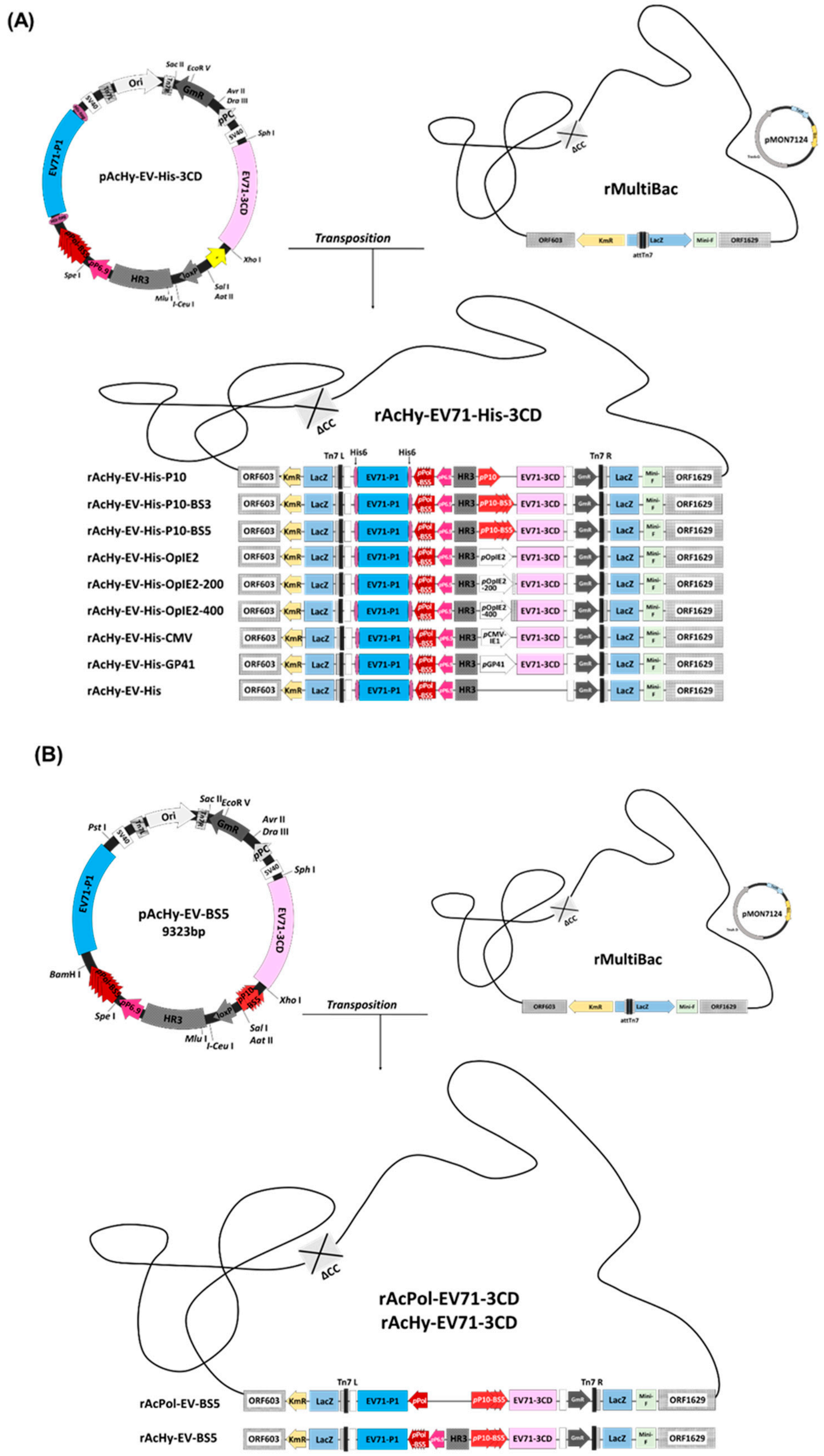

Figure S3. Schematic representation of the recombinant viruses. (A) Recombinant viruses were generated so that the P1 precursor, with His6-tag fused to both termini, was expressed by the hyperexpression vector, and 3CD was expressed by various promoters. (B) Recombinant viruses were generated to express the P1 precursor (from which the His6-tag was removed) by the hyperexpression vector or a standard vector, and 3CD was expressed by the p10-BS5 promoter.
